# Antibodies to FXa and thrombin in patients with SLE differentially regulate C3 and C5 cleavage

**DOI:** 10.1136/lupus-2022-000738

**Published:** 2022-08-25

**Authors:** Thomas McDonnell, Raj Amarnani, Carina Spicer, Hajar Jbari, Charis Pericleous, Valentina A Spiteri, Chris Wincup, Bahar Artim-Esen, Ian Mackie, Marina Botto, Anisur Rahman, Ian Giles

**Affiliations:** 1 Biochemical Engineering Department, UCL, London, UK; 2 Department of Rheumatology, University College London, London, UK; 3 Merck & Co, Kenilworth, New Jersey, USA; 4 National Heart and Lung Institute, Imperial College London, London, UK; 5 Department of Structural and Molecular Biology, University College London, London, UK; 6 Department of Internal Medicine, Istanbul University, Fatih, Turkey; 7 Department of Haematology, University College London, London, UK; 8 Imperial College Healthcare NHS Trust, London, UK

**Keywords:** Systemic Lupus Erythematosus, Antibodies, Antiphospholipid, Autoantibodies

## Abstract

**Objectives:**

The significance of antibodies directed against activated factor X (FXa) and thrombin (Thr) in patients with SLE and/or antiphospholipid syndrome (APS) is unknown. FXa and Thr are coregulated by antithrombin (AT) and activate complement. Therefore, we studied the ability of anti activated factor X (aFXa) and/or anti-(a)Thr IgG from patients with SLE±APS to modulate complement activation.

**Methods:**

Patients with SLE±APS were selected on the basis of known aThr and/or aFXa IgG positivity, and the effects of affinity-purified aFXa/aThr IgG on FXa and Thr-mediated C3 and C5 activation were measured ±AT. Structural analyses of FXa and Thr and AT–FXa and AT–Thr complexes were analysed in conjunction with the in vitro ability of AT to regulate aFXa–FXa and aThr–Thr-mediated C3/C5 activation.

**Results:**

Using affinity-purified IgG from n=14 patients, we found that aThr IgG increased Thr-mediated activation of C3 and C5, while aFXa IgG did not increase C3 or C5 activation. Structural analysis identified potential epitopes and predicted a higher likelihood of steric hindrance of AT on FXa by aFXa IgG compared with the AT–Thr–aThr IgG complex that was confirmed by in vitro studies. Longitudinal analysis of 58 patients with SLE (±APS) did not find a significant association between positivity for aFXa or aTHr IgG and C3 levels or disease activity, although there was a trend for patients positive for aFXa IgG alone or both aFXa and aThr IgG to have lower levels of C3 compared with aThr IgG alone during clinical visits.

**Conclusions:**

We propose a novel method of complement regulation in patients with SLE±APS whereby aFXa and aThr IgG may have differential effects on complement activation.

**WHAT IS ALREADY KNOWN ON THIS TOPIC**

- Currently, it is known that complement and coagulation interact, and that anti activated factor X (aFXa) and anti (a)Thr antibodies are present in both SLE and antiphospholipid syndrome.
- aFXa and aThr have been shown to have effects on calcium flux, but their role in complement activation is unknown.

**WHAT THIS STUDY ADDS**

- We show complement activation may be differentially regulated in patients with SLE by aFXa and aThr antibodies.
- Also we show that thrombin (Thr)-mediated activation of C3 and C5 was enhanced by aThr but not aFXa antibodies.
- Finally, we present data to suggest differential binding to a natural inhibitor in modelling and in vitro studies may explain these findings.

**HOW THIS STUDY MIGHT AFFECT RESEARCH, PRACTICE OR POLICY**

- Complement activation is linked to disease flares in lupus. This paper suggests aFXa may be a contributing factor to monitor in patients with low C3.
- This paper also highlights a new mechanism of action for autoantibodies targeting drug targets (activated factor X and Thr), suggesting monitoring these antibodies in patients receiving those therapies may be important.

## Introduction

Excessive activation of coagulation and complement pathways contributes to inflammatory and thrombotic manifestations of autoimmune rheumatic disease, principally SLE and antiphospholipid syndrome (APS).^1^ SLE and APS are characterised by immune dysfunction, coagulation and complement dysregulation plus autoantibody formation. Increasing evidence points towards coactivation and regulation of complement and coagulation pathways.^2^

The coagulation pathway consists of the intrinsic and extrinsic pathways leading to activation of factor X (activated factor X (FXa)), thrombin (Thr) generation, fibrin formation and haemostasis.^3^ Activation of this pathway is tightly controlled by fibrinolytic agents such as plasmin and inhibitors of serine proteases (SP), principally antithrombin (AT) III.

The complement system is a proteolytic cascade of SPs that are activated via multiple (classical, alternative and lectin) pathways converging to^4^ where C3 convertases cleave C3 to C3a and C3b to form a C5 convertase. The C5 convertase cleaves C5 to C5a and C5b, leading to production of the membrane attack complex. The central importance of C3 and C5 in the complement cascade mirrors that of FXa and Thr in the coagulation cascade, and regulatory interactions exist between these pathways.

While complement consumption is recognised to be important in disease pathogenesis, activity and damage in SLE, there have been few studies of how interactions with coagulation cascades may influence complement activation. Liang *et al*^5^ showed the combination of raised levels of D-dimers (indicating activation of coagulation cascade) and low levels of C4 performed well as a laboratory measure of SLE activity in comparison to standard markers of anti-dsDNA antibody and C3 levels. This study, however, did not establish the mechanism of coagulation–complement interaction in SLE. Interestingly, FXa and Thr have been shown to activate complement directly, without involvement of traditional pathways of complement activation.^6 7^ Furthermore, inhibition of FXa in patients with APS±SLE, with rivaroxaban (a highly selective direct FXa inhibitor), led to inhibition of complement and coagulation factors.^8^ Infact, both FXa and Thr are both controversial therapeutic targets, with rivaroxaban (FXa targeting) having shown both positive^9^ and negative outcomes^10^ in patients, while dabigatran (Thr targeting) has also been the subject of some debate,^11^ and unlike rivaroxaban, dabigitran has never shown any effects on complement activation. Therefore, increased understanding of the mechanisms of coagulation–complement interactions has the potential to improve measures of disease activity and to develop new therapeutic approaches.

In around 50% of patients with SLE and/or APS, increased levels of anti(a)Thr and antiactivated factor X (aFXa) IgG have been identified and shown to have inhibitory effects on inactivation of procoagulant SP and functional activities of anticoagulant/fibrinolytic SP.^12^ The potential effect on complement activation has not been previously addressed in vitro. Therefore, we investigated whether polyclonal IgG with FXa or Thr reactivity from patients with SLE±APS may alter the interactions of FXa and/or Thr with C3 and/or C5.

## Methods

### Patients

Patients (n=14) with APS and/or SLE, fulfilling relevant disease classification criteria,^13^ were identified from routine clinic visits at the University College London Hospital based on aFXa and/or aThr IgG positivity.^14^ Serum was stored at −20°C.

### Clinical correlations

Patients (n=58) were identified retrospectively as either aFXa positive or aThr positive. Their C3 level from their serum C3 levels from up to their last 10 consecutive clinical visits were collected to determine if historical positivity for aFXa or aThr was associated with lower levels of C3 in the clinic.

### Immunological characterisation and purification of antiserine protease IgG

Anti-FXa and aThr IgG were purified as described previously.^14^ Briefly, for purification, serum was applied sequentially to a heparin column, an immobilised SP column (FXa or Thr) and a protein-G column. Elution from the IgG column was dialysed versus Phosphate Buffered Saline (PBS) and quantified by biotinochronic assay kit. Antibodies were shown to be non-cross reactive with the other antigens when purified from a double-positive patient (data not shown).

### Measurement of complement activation

Commercial C3 or C5 (Sigma) was incubated with FXa or Thr (Cambridge Biosciences) at a 1:1 ratio for 2–4 hours at 37°C. Samples were run on a 4%–12% gel using MES buffer/Bolt System (Invitrogen). Proteins were transferred to polyvinylidene difluoride (PVDF) membrane (Amersham, 1 hour at 10 V). Membranes were blocked with 5% skimmed milk (1 hour) and incubated overnight (rolling at 4°C) in primary antibody specific for either C3 or C5 (Abcam, 3 μL in 1666 dilution). Secondary antibody (anti-mouse Horse Radish Peroxidase (HRP)-conjugated antibody, Dako) was incubated with the membrane for 1 hour at room temperature before washing (0.1% PBS-Tween, 4×15 min) and exposure with ECL Prime (Amersham) for 30 s. Densitometry was performed using Analysis One software.

### Effect of affinity-purified anti-SP antibodies on complement cleavage in the presence or absence of AT

Anti-SP antibodies were preincubated at a molar ratio of 1:1 with either FXa or Thr for 1 hour at 37°C before addition of C3 or C5 for 4 hours at 37°C and cleavage to C3a or C5a measured as detailed previously.

Optimal AT (Cambridge Bioscience) inhibition of C3 cleavage by FXa and Thr occurred at a 2:1 M ratio of AT to enzyme in the absence of heparin (data not shown). Therefore, the effect of anti-SP antibodies on cleavage of C3 or C5 was repeated with AT at a 2:1 M ratio.

### Molecular dynamics and epitope prediction

Structures of FXa and Thr were generated from 2BOH and 1AHT, respectively. Structures were prepared for molecular dynamics analysis using Glycan Reader and Modeller at the CHARMM-GUI website (http://www.charmm-gui.org/). Molecular dynamics were run as mentioned previously^15^ using CHARMM36 force field. Convergence of the simulations for all systems was checked through the comparison of average root mean square deviation (RMSD) using the Visual Molecular Dynamics programme (VMD). Epitope prediction was carried out using Discotope V.1.1 using the FXa and Thr PDB files (PDB ID: 2BOH and 1AHT). Each simulation was repeated three times and the data were averaged for analysis. The predicted sites where then modelled onto resolved crystal structures of the FXa/AT and Thr/AT complexes.

### Statistical analysis

Data are presented as means±SE from the mean (SEM) and were analysed in GraphPad Prism V.8.0 using t-tests and one-way analysis of variance as appropriate. Normality was derived using Kolmogorov-Smirnov testing. Differences between means with a p value of <0.05 were considered significant.

## Results

### Clinical and laboratory features

All purified patients (n=14) were female; 2 patients had APS and 13 had SLE, while the average age was 53 years (range 26–82). Average antibody levels were as follows: anti-cardiolipin IgG (aCL) 30 GPLU (mean 30.04 SD 24.3 GPLU), aB2GPI 14.5 GBIU (14.63 GBIU, 6.43 GBIU), aFXa 33 U (33.01 U, 25.89 U), aThr 35 U (35.22 U, 33.79 U). A total of four patients were lupus anticoagulant (LA) positive; two patients were isolated LA positive, without clinical manifestations of APS tested at a single timepoint. The most common symptoms in patients were rashes (8/14), joint pain (13/14), renal (7/14), serositis (7/14) and central nervous system lupus (5/14). A wider cohort of n=58 patients showed (demographics in online supplemental table 1) no significant clinical association was seen between levels of positivity for either aFXa or aThr antibodies and low C3 levels or disease flares (online supplemental figure 1). A trend however, was observed for lower C3 levels in double-positive (aFXa and aThr IgG) and single-positive (aFXa) patients compared with aThr positive alone. Median values of C3 were below clinical threshold (0.9 g/L) for 46% of double-positive patients in comparison to 36% for aFXa IgG-positive patients and 15% for aThr IgG-positive patients. A similar pattern was observed in the percentage of patient visits with clinically low C3 levels, a marker of active disease; 44% of visits for double-positive patients were below 0.9 g/L compared with 28% for aFXa IgG-positive and 25% for aThr IgG-positive patients.

10.1136/lupus-2022-000738.supp2 Supplementary data

10.1136/lupus-2022-000738.supp1 Supplementary data

### Antiserine protease antibodies affect FXa and Thr-mediated complement activation

**Figure 1:**
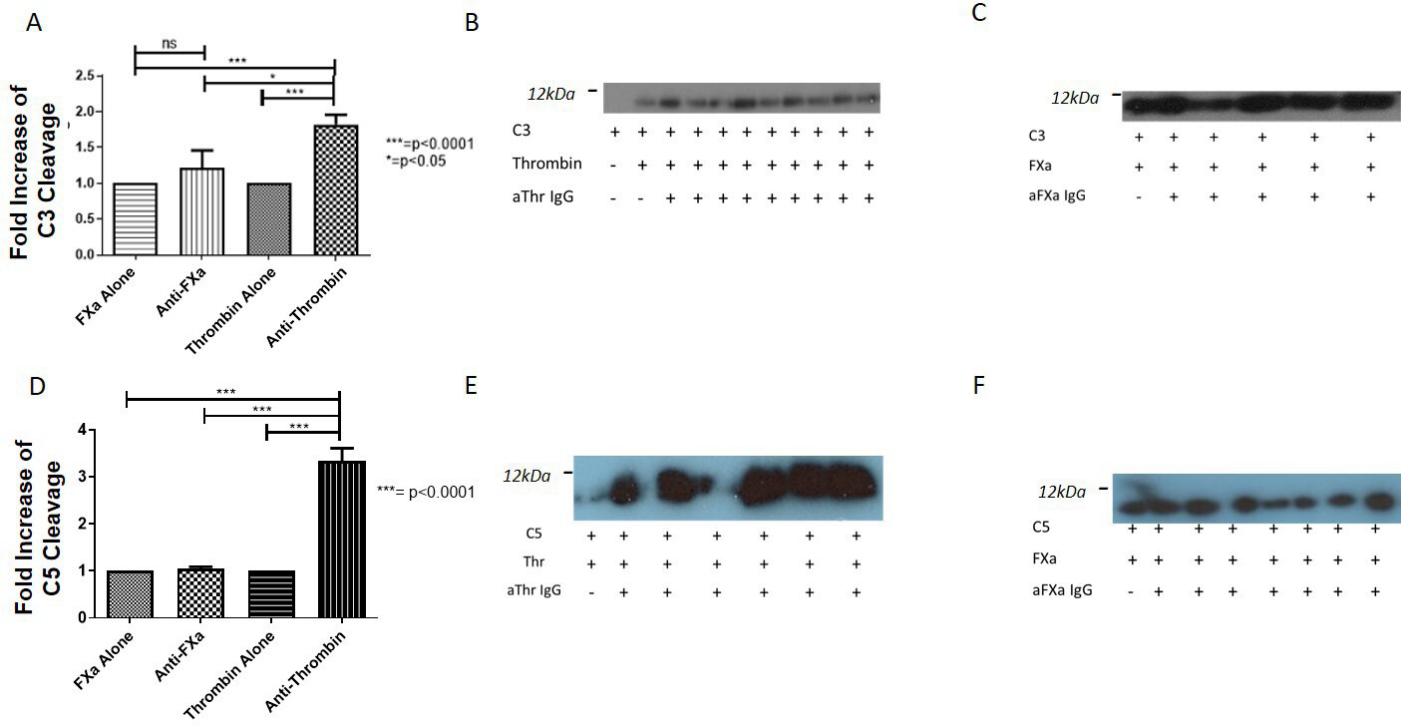
Enzymatic cleavage of C3 and C5 by FXa and Thr. The effect of purified IgG on FXa and Thr-mediated C3 and C5 activation was measured by the quantification of the cleavage products, either C3a or C5a. (A) Preincubation of either (n=6) aFXa IgG to FXa or (n=8) aThr IgG to Thr leads to increased cleavage of C3 with Thr than FXa (1.8-fold vs 1.3-fold, p=0.01). Associated immunoblots (B, C), cleavage in the presence of aThr IgG was significantly higher than for aFXa IgG. (D) C5 cleavage is unchanged by the addition of (n=6) aFXa IgG, while (n=6) aThr IgG significantly increases the cleavage of C5 by threefold (p≤0.0001, ANOVA), demonstrated in immunoblots (E, F) showing large differences in E (aThr IgG), while there is relatively little difference in F (aFXa IgG). Statistical significance was seen for all groups in comparison to Thr+aThr IgG (p<0.001, ANOVA). A representative immunoblot is included showing the significant increase in C5 cleavage due to the addition of aThr antibodies. Representative full-sized westerns are available on request. aFXa, antiactivated factor X; ANOVA, analysis of variance; FXa, activated factor X; ns, not significant; Thr, thrombin.

After preincubation of antigen and antibody, C3 cleavage was significantly increased by aThr IgG only (p=0.0003) compared with aFXa IgG (figure 1A–C). The increase with aThr antibodies was significantly higher than that with aFXa antibodies (p=0.01, figure 1A). Preincubation with anti-Thr IgG significantly increased Thr-mediated C5 activation threefold (p≤0.0001, figure 1D, E), while preincubation with aFXa IgG had no effect on FXa-mediated C5 cleavage (p=0.9, figure 1D, F). Given that aThr increased C3 and C5 cleavage, while aFXa had no effect on C3 or C5 cleavage, we hypothesised the differential effects of these IgG maybe due to their interactions with target SP and carried out molecular modelling studies.

### Molecular modelling identifies epitopes on FXa and Thr, predicting steric hindrance of AT

**Figure 2:**
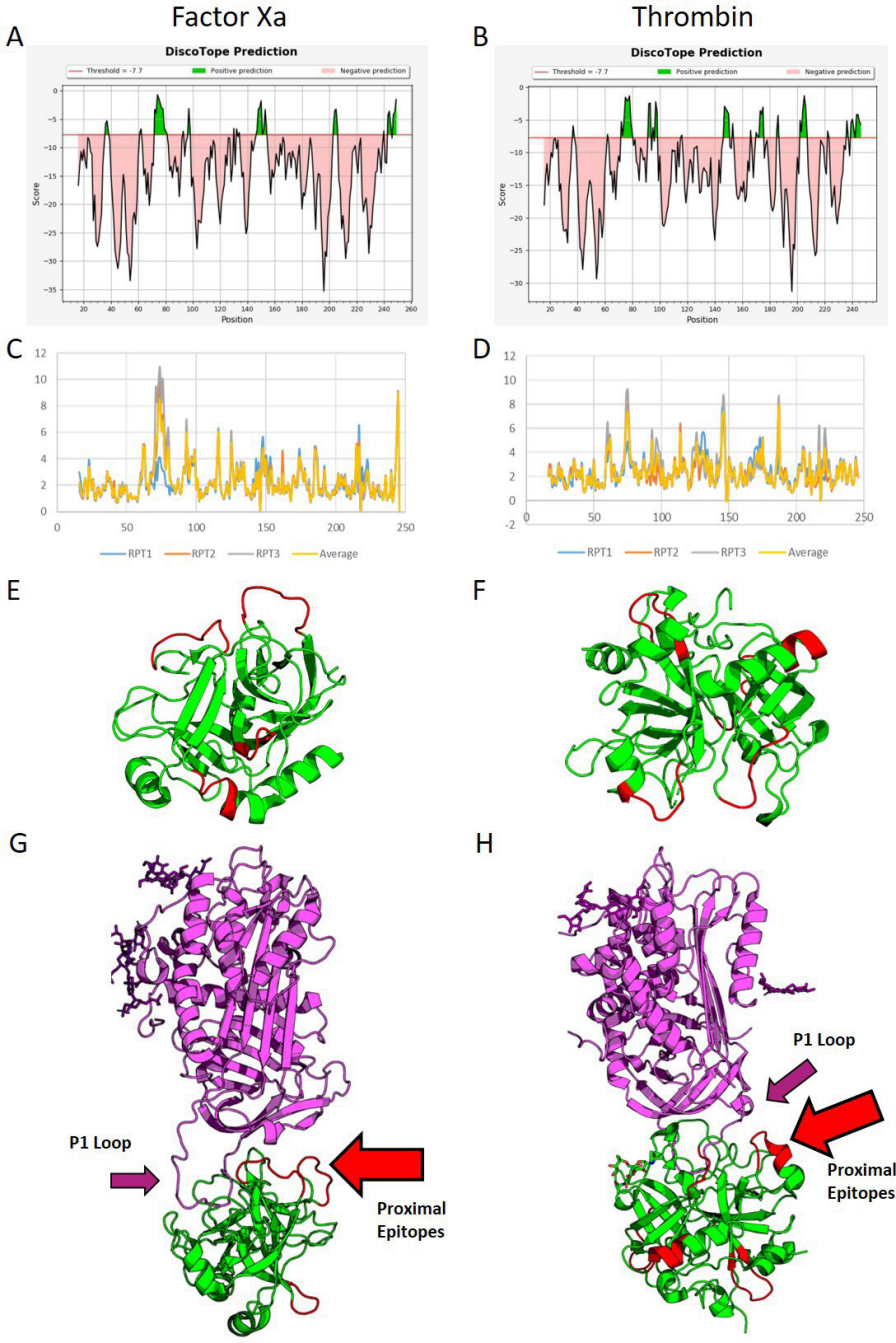
Molecular simulation and in silico epitope prediction. (A, B) Epitope prediction scores from Discotope for FXa and Thr, respectively. Green represents a 75% sensitivity for the associated amino acids being found within epitope sites. More sites are predicted for Thr than FXa. (C, D) RMSD for the backbones of both proteins, FXa (C) and Thr (D). Large peaks in flexibility are associated with epitope sites; as can be seen in FXa, there are fewer peaks in flexibility. The largest peak is found between residues 65 and 82. Smaller, more frequent peaks are seen for Thr. These peaks were cross-matched with the scores from Discotope and visualised using PyMOL on the proteins (E, F) (FXa and Thr, respectively) with potential epitopes in red and other protein sequences in green. Finally, these models were matched against crystal structures for the binding of AT3 (G, H). The purple ribbons represent AT, while the green represent FXa and Thr, respectively; the red highlighted sections represent potential epitope sites. The P1 loop is highlighted by the purple arrows. As can be seen, two of the potential epitope sites for binding in FXa align with the binding site for AT (2/4 sites, G, red arrow), while only one of the potential epitope sites is proximal to the site in Thr (1/6 sites, H, red arrow). AT, antithrombin; FXa, activated factor X; Thr, thrombin.

The molecular dynamic simulations identified several potential epitopes within each protein (highlighted in green, figure 2A, B). RMSD analysis showed several significant peaks in conformational flexibility (figure 2). Cross-referencing the Discotope and RMSD outputs generated four potential epitope sites on FXa and six on Thr (figure 2E, F). The proximity of these epitopes to AT binding sites was assessed on crystal models (PDB ID: 2GD4 and 1TB6). In FXa, two of the potential antibody epitope sites face the AT binding site (2/4, 50%), while for Thr, only one potential epitope site was proximal to the AT binding site (1/6, 16%) (figure 2G, H). Therefore, we hypothesised that aFXa IgG may have a greater inhibitory effect on AT than aThr IgG.

### Anti-SP IgGs have differential effects on AT-mediated inhibition of coagulation–complement activation

**Figure 3:**
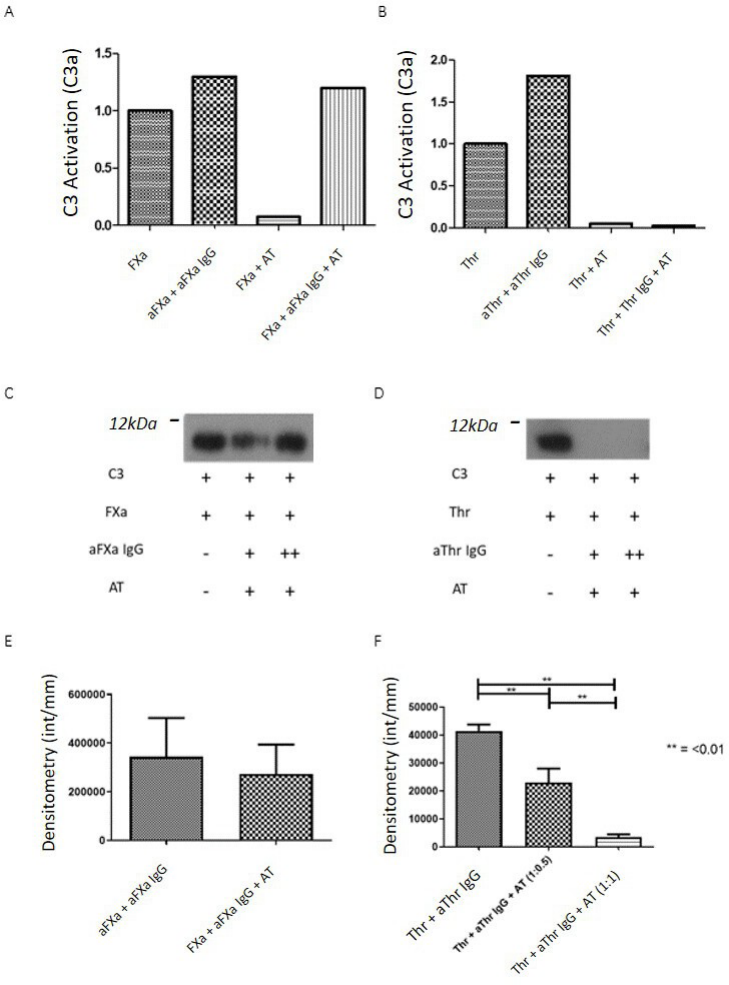
Addition of AT III to antibody–antigen interactions. (A, B) Effect of AT on FXa (A) and Thr (B) mediated C3 cleavage measured by the quantification of the cleavage products, either C3a or C5a. aFXa IgG preincubated with FXa can overcome the effect of AT on FXa but not Thr. At was capable of inhibiting Thr even in the presence of aThr IgG. This finding is demonstrated in the immunoblots (C, D) showing a significant though not complete reduction of cleavage for FXa before restoration of activity in increased antibody doses, while for aThr, no cleavage is seen in any sample containing AT. (C, D) The effect of AT on C3 cleavage by FXa (C) and Thr (D) in the presence and absence of AT is shown. (E, F) AT fails to inhibit the activity of FXa in the presence of aFXa IgG in a C5 cleavage experiment; however, C5 cleavage in the presence of aThr is inhibited significantly (F) in a dose dependent manner (p<0.01). Demonstrative full-sized westerns are available on request. aFXa, antiactivated factor X; AT, antithrombin; FXa, activated factor X; Thr, thrombin.

We examined whether this differential relationship between potential anti-SP epitopes and AT binding site on FXa and Thr alters the ability of AT to inhibit FXa and/or Thr-mediated C3/5 activation in the presence of anti-SP antibodies. FXa and Thr-mediated C3 activation was completely inhibited by a 1:1 ratio of AT (figure 3A, B). Preincubation of aFXa IgGs with their antigen in the presence of AT did not inhibit FXa-mediated cleavage of either C3 (figure 3A, C) or C5 (figure 3E). In contrast, aThr IgG had no effect on the ability of AT to abrogate cleavage of C3 by Thr (figure 3B, D) or of C5 by Thr at a molar ratio of AT to Thr of 1:1 and was dose dependent (figure 3F).

## Discussion

We found that FXa and Thr mediated cleavage of C3, and C5 was increased by aThr but not aFXa IgG. Using molecular modelling, we identified potential sites of aSP epitopes on FXa and Thr, with an increased percentage of potential epitopes being found close to the AT binding site on FXa (50%) in comparison to Thr (16%). This increased clustering of aFXa and AT compared with aThr and AT binding sites means that aFXa may increase steric hindrance of AT on FXa compared with aThr and AT on Thr and would explain why AT-mediated inhibition of FXa was overcome by aFXa, but AT-mediated inhibition of Thr by cognate IgG from patients with SLE±APS was not. The exact epitope site, however, requires delineation in future work. Although clinical correlation did not find any significant associations between aSP positivity, C3 levels and disease activity, there was a trend for patients positive for aFXa IgG alone or with aThr IgG to have lower levels of C3 during clinical visits.

Complement consumption is an established marker of disease flare in SLE; similarly altered C5a levels have been shown in both SLE nephritis and neuropsychiatric events.^16^ There are few studies, however, of coagulation–complement interactions in SLE, although correlations between the two cascades have been shown.^5^ The link of complement and coagulation is better shown in APS, where in clinical trials of rivaroxaban (an anti-FXa therapy) reduced C3a and C5a levels.^8^ Furthermore, a recent study showed a regulatory relationship between FXI and complement factor H, coregulating the activation of both cascades, with a downstream effect on CD55 in an endothelial model.^17^

The clinical relevance of our findings are uncertain. The non-significant trend for patients positive for aFXa IgG alone or both aFXa and aThr IgG to have lower levels of C3 during clinical visits may indicate that aFXa IgG may contribute to decreased C3 levels in patients, and prospective analysis with direct correlation of aFXa and aThr IgG and C3 levels at each visit is required to explore this relationship. Other limitations include low numbers of patients with APS in our purified IgG analysis and that we have not confirmed epitopes by peptide mapping or measured IgG subclass. In addition, we cannot be certain that we have used physiological levels of FXa and Thr as they are rapidly complexed to inhibitors, although increased local concentrations are seen at the site of cellular damage of activation. Similarly, although some APS-positive patients are included, due to the low number, it is hard to translate this research into patients with APS as a whole.

In conclusion, we present a novel mechanism by which complement activation may be achieved and differentially regulated in patients with SLE and potentially APS through aFXa and aThr antibodies while evading natural regulatory mechanisms. Further research is required to fully characterise this mechanism and its therapeutic potential, as well as the effect these antibodies may have on efficacy of direct FXa and Thr inhibitors if used in these patients.
