# Supplementary material for: Antibodies to FXa and thrombin in patients with SLE differentially regulate C3 and C5 cleavage
Source: Lupus Sci Med. 2022 Aug 25;9(1):e000738. doi: 10.1136/lupus-2022-000738 (PMC9422842; doi:10.1136/lupus-2022-000738)
Supplement: Supplementary data [file lupus-2022-000738supp002.pdf]

Tables

	**SLE/APS**
Age (SD)	46.29 (12.46)
Sex Female (Male)	55 (3)
Manifestations SLE, (n)	Mucocutaneous (54),<br>CNS (7),<br>Renal (17),<br>Haem (3),<br>Joint (57),<br>Serositis (16)
Manifestations APS, (n)	VT (4),<br>PM (2),<br>CVA (2)
aCL, n (mean, SD)	8 (17.91, 42.03)
Anti-β2GPI, n (mean, SD)	3 (14.15, 9.87)
LA positive, n	16
Other autoantibody positivity (n)	aDNA (39),<br>Sm (12),<br>RNP (24),<br>Ro (28),<br>La (3),<br>RF (9),
aFXa, n (mean +/- SD)	38 (21.20, 16.67)
aThr, n (mean +/- SD)	44 (32.43, 26.20)

Supplimentary Table 1. Patient demographics of n=58 clinical associations cohort
