# Supplementary figures and images for: Antibodies to FXa and thrombin in patients with SLE differentially regulate C3 and C5 cleavage
Source: Lupus Sci Med. 2022 Aug 25;9(1):e000738. doi: 10.1136/lupus-2022-000738 (PMC9422842; doi:10.1136/lupus-2022-000738)

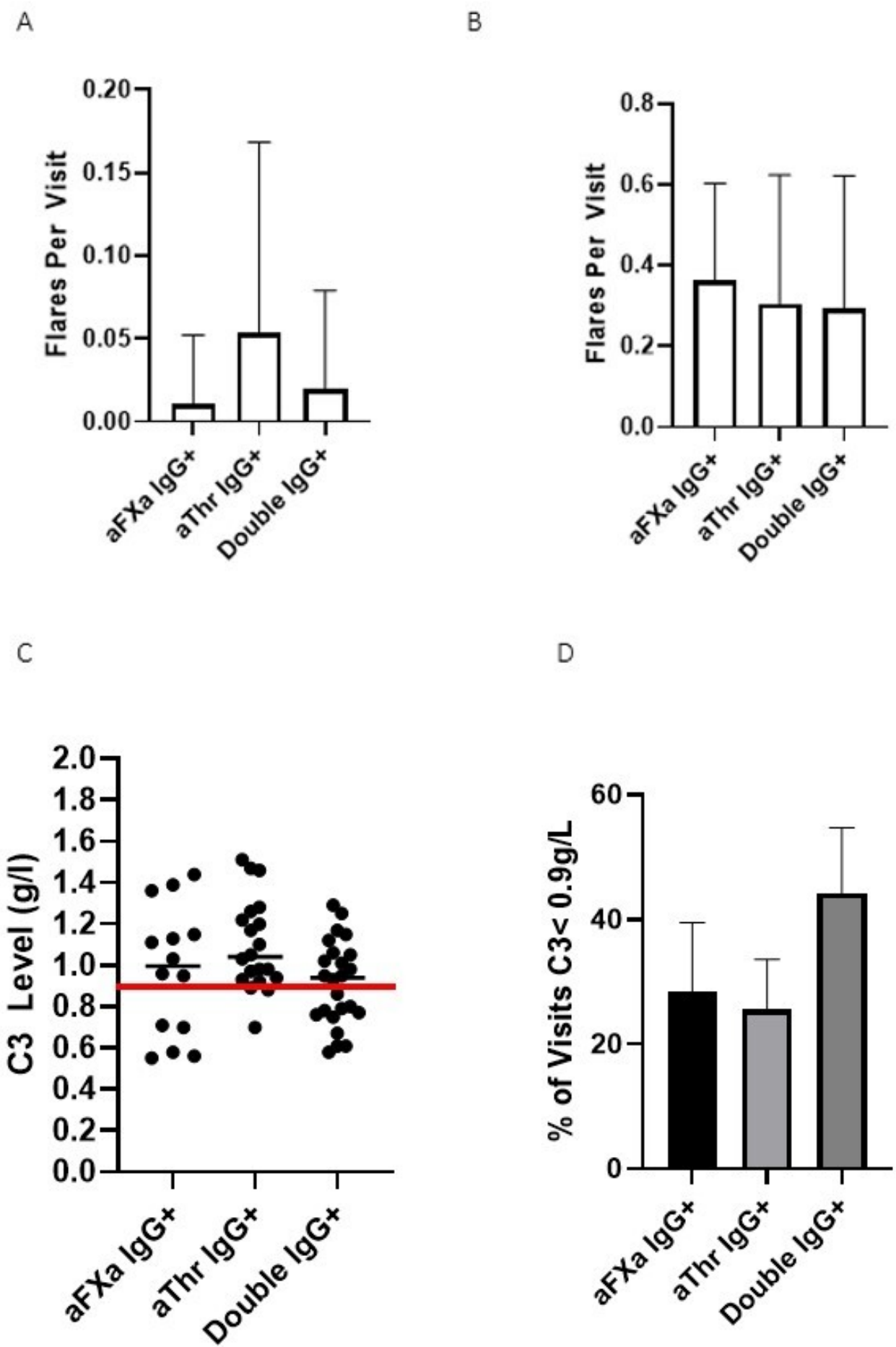

Supplement: Supplementary data [file lupus-2022-000738supp001.pdf]
